# Anthropogenic noise, but not artificial light levels predicts song behaviour in an equatorial bird

**DOI:** 10.1098/rsos.160231

**Published:** 2016-07-06

**Authors:** Adriana M. Dorado-Correa, Manuel Rodríguez-Rocha, Henrik Brumm

**Affiliations:** 1 Communication and Social Behaviour Group, Max Planck Institute for Ornithology, Seewiesen, Germany; 2 Foundation Chimbilako, Carrera 34 # 10–77, Bogotá, Colombia

**Keywords:** dawn song, light pollution, noise pollution, rufous-collared sparrows, tropics, *Zonotrichia capensis*

## Abstract

Birds in cities start singing earlier in the morning than in rural areas; commonly this shift is attributed to light pollution. Some studies have suggested that traffic noise has a stronger influence on singing activity than artificial light does. Changes in the timing of singing behaviour in relation to noise and light pollution have only been investigated in the temperate zones. Tropical birds, however, experience little seasonal variation in day length and may be less dependent on light intensity as a modifier for reproductive behaviours such as song. To test whether noise or light pollution has a stronger impact on the dawn chorus of a tropical bird, we investigated the singing behaviour of rufous-collared sparrows (*Zonotrichia capensis*) in Bogota, Colombia at two times during the year. We found that birds in places with high noise levels started to sing earlier. Light pollution did not have a significant effect. Birds may begin to sing earlier in noisy areas to avoid acoustic masking by traffic later in the morning. Our results also suggest that some tropical birds may be less sensitive to variations in day length and thus less sensitive to light pollution.

## 1. Introduction

The world is facing rapid environmental changes as a result of ever-increasing urbanization. The growth of urban areas is projected to attain unprecedented levels in the next few decades, which will have dramatic effects on ecosystems; including the extinction of species [1]. In order to adjust to urban environments animals often need to change a whole suite of traits, including behaviour, physiology and morphology. This is particularly true for birds, because many avian species colonize urban areas [2]. Owing to anthropogenic activities these areas are characterized by fundamental changes in ecological factors, e.g. habitat fragmentation, changes in microclimate, limitation of resources, alteration of resources flow, changes in species interactions, and pollution [3].

Indeed, urbanization is considered one of the major causes of pollution around the world [4], including chemical contamination and interference by noise and light emissions. In birds, light pollution is often associated with changes in biological rhythms [5–7], which can ultimately affect breeding behaviours and fitness. For example, blue tits (*Cyanistes caeruleus*) exposed to artificial light during the night lay eggs earlier than individuals living in darker areas [8]. Changes in avian reproductive success have also been linked to noise pollution; it has been observed that birds in noisy areas have reduced numbers of offspring [9,10] or show lower rates of provisioning for their young [11]. Bird reproduction is particularly susceptible to noise because most species rely on acoustic signals (songs) to defend territories and attract mates [12]. Therefore, acoustic masking by noise is likely to have major fitness consequences [13]. Likewise, there is a growing body of evidence showing that birds adjust their song characteristics and their song performance to mitigate interference from noise [2]. For instance, in response to increases in the background noise level, birds increase the amplitude of their songs, and additionally they may also adjust the redundancy, the duration, the frequencies and the timing of their vocalizations (reviewed in [14]).

The daily singing activities of many songbirds have a peak just before sunrise, a behaviour that is commonly referred to as the dawn chorus [12]. While the function of the dawn chorus is still debated, several hypotheses suggesting its adaptive value have been proposed. For instance, singing may be more profitable than foraging before sunrise because the movement of invertebrates at that time is reduced due to low temperatures and light levels [15–17]. It may also be that the microclimatic conditions before sunrise create conditions that are particularly favourable for sound transmission because of low acoustic attenuation [18]. In addition, some studies have proposed that the onset of dawn singing may be an indicator of male quality [19,20] or age [21] and thus could be linked to female choice. Indeed, the significance of the dawn chorus for reproduction is highlighted by several studies which found that males that started singing earlier in the morning had more extra-pair offspring [8,22,23]. Corroborating evidence for this notion comes from a recent study demonstrating that an experimentally delayed dawn song was associated with a decreased number of extra-pair offspring in great tits (*Parus major*) [24].

The onset of the dawn chorus is affected by light pollution: birds start to sing earlier in the morning [5,8,25,26] and earlier in the year [27] when they are exposed to artificial light during the night. Some authors have argued that noise pollution also has a crucial impact on the timing of the dawn chorus. For example, Nordt & Klenke [28] found that blackbirds (*Turdus merula*) started the dawn chorus earlier in areas with high noise levels. In another study, starlings (*Sturnus unicolor*) and house sparrows (*Passer domesticus*) advanced their dawn singing in response to noise playback [29]. Furthermore, European robins (*Erithacus rubecula*) were found to shift their singing activity to the night in urban areas, presumably to avoid high levels of diurnal background noise [30]. All these studies have been conducted in temperate zones, where the breeding stages of birds are tightly linked to photoperiod. In the tropics, however, the effects of light and noise pollution on bird behaviour may be different because day length varies only marginally and some species seem not to rely on day length cues to time their reproductive stages [31]. Even though some tropical species have the capacity to respond to photoperiodic changes in principle [32], it may be that they do not use it in their natural habitats either because variations in day length are too small, or because day length is not a reliable predictor of environmental conditions. Therefore, it is unclear whether light pollution affects the timing of reproductive behaviours, such as song, in tropical birds as it does in temperate birds. This issue is important because it will reveal whether the patterns of the urban ecology of bird song observed so far reflect general processes or whether they are specific to temperate conditions where photoperiod triggers reproduction. The neglect of studies on tropical birds is particularly profound since not only are many of the world's largest urban areas found in the tropics, but the tropics are also the regions with the highest bird biodiversity [33,34].

The main goal of this study was to disentangle whether anthropogenic noise after dawn or artificial light during the night better predicts the onset of the dawn chorus in rufous-collared sparrows (*Zontrichia capensis*) in Bogota, Colombia, one of the largest cities in tropical South America. Rufous-collared sparrows are widespread in the Americas, ranging from Southern Mexico to the temperate zone in Tierra del Fuego [35,36]. In tropical areas, they do not rely on photoperiod to regulate their life stages, but they breed when local conditions and weather are favourable [31,37]. In the Colombian Andes, rufous-collared sparrows reproduce throughout the entire year, with breeding peaks in mid-January and mid-June in certain areas [38]. In Bogota, rufous-collared sparrows are present within the city and its surroundings, and their dawn chorus can be heard throughout the year (M Rodríguez-Rocha 2012, unpublished data). We chose different sites in the metropolitan area to include habitats with different combinations of light and noise pollution and we related these variables to the onset of the dawn chorus. Because we assumed a weaker link between diel behaviours, such as the onset of dawn singing, and photoperiod under the almost invariable day-length conditions in our study population, we expected noise to have a stronger impact on the onset time of dawn singing than light pollution.

## 2. Material and methods

### 2.1. Study site

We studied 33 urban sites in public parks and gardens in the city of Bogota, Colombia (4°35'53^″^ N, 74°4'33^″^ W). Bogota is located in the tropical zone; around 500 km north of the equator and sunrise times vary by about 21 min throughout the year. Buildings or houses surrounded all the places of the study, creating green islands inside the urban area. Eighteen of these were visited in May–June 2013 and 15 in November–December 2013. In addition, six of the 18 May–June sites were visited again in November 2015. All of the sites were chosen based on a previous monitoring study [39] that indicated where rufous-collared sparrows are present. The two sampling seasons were chosen because they both coincided with the end of the rainy season in the particular years, during which time similar precipitation and temperature conditions were experienced. We selected these sites with particular attention to cover a broad range of different combinations of daytime noise and light pollution levels (see the electronic supplementary material, figures S2 and S3). The average distance between urban sites was 1.5 km (minimum 100 m). At every site, we recorded the onset of the dawn chorus of rufous-collared sparrows, the levels of light pollution and the levels of daytime noise. Daytime noise was mainly due to traffic, and the light pollution was due primarily to street lamps. To compare the onset of the dawn choruses of urban birds to those of rural birds, we also collected the same data from five rural areas in May–June. The rural sites were located between 120 and 125 km from Bogota, in areas with very low levels of noise and light pollution. The average distance between them was 1.5 km (minimum 500 m).

### 2.2. Onset of dawn chorus, light and noise pollution levels

Each morning, at 3.50 h or earlier (i.e. at least 10 min earlier than the earliest record of the onset of the dawn chorus of this species in Bogota), we visited sites and recorded the time when we heard the first individual singing (see the electronic supplementary material, figure S1 for rufous-collared sparrow song and exemplary traffic noise recordings). We collected data on weekdays from Monday to Friday to ensure comparable levels of noise due to morning commuting traffic. We marked the position where the bird first sang and then took the measurements of light and noise as close as possible to this position. The height of the song perches ranged approximately between 0.5 m and 4.0 m from the ground. Night-time light levels were measured with a digital photometer (Voltcraft MS 1500, Germany) between 80 and 160 min after sunset. Five light measures were taken for 10 s each, four of them with the photometer held with a stretched-out arm in four directions separated by 90° and one measurement with the metre pointing upwards. With the same protocol, noise levels were taken, using a sound pressure level (SPL) meter (Voltcraft SL 400, Germany) during the peak traffic hours after sunrise, from 06.00 to 08.30. This time period was chosen based on a traffic census made by the Secretaria Distrital de Mobilidad of Bogota. We measured noise levels every 10 s for 1 min pointing the SPL meter in a different direction. Noise levels were measured as dB(A) SPL, a procedure that measures the noise in decibels relative to the standard reference of sound pressure in air, 20 µPa. Twenty micropascals correspond to 0 dB SPL, which is the threshold of human hearing. The frequency range of the SPL meter was 31.5 Hz–8 kHz and the reading range was 30–130 dB(A). The SPL meter and the photometer were held approximately 1.25 m above the ground.

### 2.3. Data analyses

The statistical analyses were carried out using R v. 2.11.0 [40]. We confirmed the normality of the data by conducting a residual analysis, plotting the residuals against the fitted values, the normal quantile–quantile (qq)-plot of the residuals, the square-root of the absolute values of the standardized residuals versus the fitted, and the residuals against the leverages. We used linear models from the R package ‘arm’ [41] to examine the relationship between noise and light levels as well as the relationship between onset time of the dawn chorus and light and noise. The onset time of the dawn chorus was calculated using the difference between the time of sunrise and the time of singing at each site. Our response variable was the onset time of the dawn chorus and the independent variables were noise levels (dB(A) SPL), light levels (lux) and period of the year (May–June; November–December). We ran the same model including daily temperature and precipitation (extracted from a meteorological database [42]) but we did not find a statistically significant relationship between the weather variables and the dawn chorus onset nor a significant interaction with the other variables. We also did not find an interaction between noise and light variables when running the model with the interaction included. Thus, we only present models excluding weather and the interaction between noise and light. In addition, we ran a linear model to compare the onset of dawn chorus in rural areas and urban areas inMay–June.

## 3. Results

The city sites covered a wide range of different levels of artificial light at night (0.7–9.4 lux; mean: 3.8 ± 2.5) and of daytime noise levels (46–75 dB(A) SPL; mean: 57 ± 10). In this sample, the noisiest site was located about 20 m from a dual carriageway and the quietest site was located in a garden in a suburban area (see the electronic supplementary material, figure S3 for recording locations). The most heavily lighted site was illuminated by several street lights within about 2 m distance, whereas the darkest site had no artificial lights in direct view. Often light and noise levels covary in cities because busier streets tend to have more lighting. However, as we systematically chose locations with different combinations of light and noise, the two measures were not correlated in our dataset (Pearson correlation: 0.14; *n* = 33, *p* = 0.41). Also there was no significant interaction between light and noise levels according to the initial model (*t* = −0.278, *p* = 0.78317, *r*^2^ = 0.73, d.f. = 27; electronic supplementary material, figure S2).

**Figure 1.**
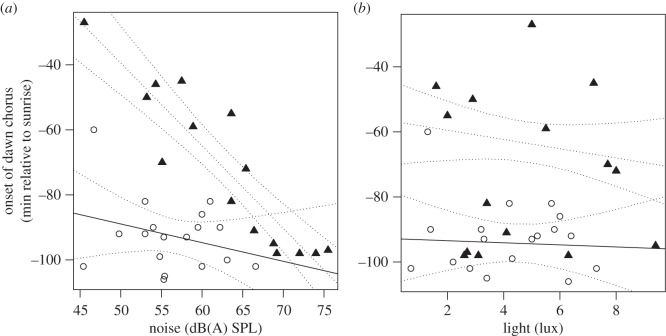
Relation between onset time of the dawn chorus of urban rufous-collared sparrows and (*a*) noise levels and (*b*) light pollution levels, May–June (circles) and November–December (triangles). Regression lines denote linear model estimation with 95% confidence intervals (dotted lines).

The onset of the dawn chorus was significantly related to daytime noise levels but the strength of this relationship depended on the season (*t* = −3.43, *p* = 0.001, *r*^2^ = 0.73, d.f. = 27, table 1 and figure 1*a*). In November–December, birds began to sing earlier in the morning in noisier places, with an average advancement of 10 min for every 5 dB increase in noise. During the May–June period, however, the same increase in noise yielded only an average advance in the dawn chorus of 2.5 min. The much stronger effect in November–December was linked to a general difference in singing behaviour between the two seasons, as birds started singing later in November–December compared to the May–June period (*t* = 3.78, *p* < 0.0001, *r*^2^ = 0.73, d.f. = 27; table 1). This seasonal difference between sites was also confirmed within sites when we resampled seven of the sites that had initially been visited in May−June. Birds did not occupy three of them and in four of them we found that birds started singing later in relation to sunrise in November than in May−June (mean November: 67.75 ± 27.62 min before sunrise; mean May−June: 91.75 ± 9.60 min before sunrise). In contrast to the observed effects of daytime noise, the onset of the dawn chorus in urban areas did not vary significantly with light pollution levels (*t* = 0.22, *p* = 0.82, d.f. = 27; table 1 and figure 1*b*).

**Table 1.** Outcome of a linear model testing the effects of light pollution, daytime noise level, and the period of the year (November–December versus May–June) on the onset of the dawn chorus in rufous-collared sparrows.

predictors	estimate	s.e.	*t*-value	*p*-value
intercept	−92.825	7.001	−13.257	<0.001
light	−0.320	1.413	−0.226	0.822
noise	−0.533	0.493	−1.079	0.289
period of the year	35.726	9.458	3.777	<0.001
light : period of the year	−0.913	1.827	−0.500	0.621
noise : period of the year	−2.037	0.594	−3.426	0.001

In comparison to the urban birds in our study, the birds in rural areas started to sing later in relation to sunrise (*t* = 3.85, *p* = 0.001, *r*^2^ = 0.79, d.f. = 19; electronic supplementary material, figure S4). On average, the rural dawn chorus began 50 min before sunrise in May−June, which is 10 min later than the latest dawn chorus onset in the city in the same period. As expected, rural levels of noise (mean = 39 ± 7 dB(A) SPL) and light (mean = 0 ± 0 lux) were low compared with the ones measured in the city (electronic supplementary material, figure S4), but this difference was not statistically significant (noise levels: *t* = −1.14, *p* = 0.27, d.f. = 19; light levels: *t* = −0.34, *p* = 0.73, d.f. = 19).

## 4. Discussion

We found that the onset of the dawn chorus of rufous-collared sparrows in a tropical city varied with the level of noise pollution. At sites with high levels of daytime traffic noise, birds started to sing earlier in the morning. By contrast, the onset of dawn singing was not correlated with levels of artificial light.

The impact of light and noise pollution on animal behaviour has been studied almost exclusively in temperate zones. Here we report for the first time how the usage of mating signals (song) varies with these anthropogenic factors in the tropics, where the photoperiod varies very little across the year. In the temperate zones with the marked seasonal changes in day length, photoperiod is a cue for the timing of reproductive stages of birds in general and for the onset of the dawn chorus in particular [43–46]. On a seasonal level, light at night can advance the timing of breeding (e.g. gonad development [47]), and the likely underlying mechanism is a perceived increase in day length [7]. Related to this, dawn singing would emerge earlier in the season through an earlier entry into the reproductive stage in areas with artificial light at night [27]. In addition, light also directly influences the onset time of dawn chorus in the morning, as demonstrated for several songbird species in the temperate latitudes [5,8,26]. However, it has been suggested that anthropogenic noise may also affect the daily onset of the dawn chorus. Birds would increase the periods for undisturbed singing by shifting their song activity to earlier hours [28,29]. In line with this notion, it has been demonstrated that two of six species tested, advanced their dawn chorus when exposed to noise [29]. On the other hand, a recent study [48] proposed that the observed shifts in singing activity in urban birds is due to light pollution rather than traffic noise; although the study did not measure noise levels directly.

Our evidence pointed to a stronger impact of noise over light pollution on the singing behaviour of an urban bird in the tropics. Rufous-collared sparrows probably sang earlier to avoid the peak of traffic noise, which occurred after dawn. Birds and other animals use a whole suite of vocal changes to communicate in noisy conditions (reviewed in [14]); most of them, however, are based on immediate individual plasticity in response to changes in the background noise. The phenomenon described here is different, because the birds adjusted their dawn song onset in anticipation of a change in the environment that occurred roughly 2 h later when the morning rush hour set in. This raises the question about the mechanisms by which this adjustment is achieved. This issue can be addressed by experiments on urban and rural birds [47] and also by studying the dawn song within cities, comparing the song onset of individual birds during weekdays with heavy commuting traffic and less noisy weekend days [49].

In contrast to previous work on temperate birds, we found that the onset of the dawn chorus did not vary with levels of artificial light at night. This finding cannot be accounted for by a difference in light pollution, as the light intensities at night measured in our study are within the range of values observed in a temperate study that found a strong effect of light [48]. Moreover, in our dataset from November–December, city birds in the least illuminated places started their dawn singing at comparable times as their conspecifics at non-illuminated rural sites.

Our findings could be explained if sunrise is not a strong seasonal cue for some birds close to the equator. In the tropics, the photoperiod changes very little across the year, unlike in the temperate zones where a marked increase in day length during spring triggers the breeding season of birds. Similarly, it has been found that changes in day length are not used as cues to trigger reproductive behaviours in tropical rufous-collared sparrows [31]. Close to the equator, some birds may be generally less entrained with the photoperiod. Therefore, disturbances of natural light regimes through light pollution may have less of an effect on their behaviour. However, to generally establish such a difference between birds in the tropics and the temperate zones, more species close to the equator need to be studied.

Interestingly, we found a seasonal difference in the onset of the dawn chorus: birds started singing later in relation to sunrise in November–December than in May–June. We could corroborate this finding by resampling some of the sites that were initially sampled in May–June again in November. The later song onset in November–December means that the birds had less time for their displays before the rush hour set in. Our data suggest that the rufous-collared sparrows shifted the onset of their singing activity to earlier periods to gain more time before their songs were masked by the traffic noise later in the day. As a result, in November–December the birds in the noisiest city areas started singing on average about 70 min earlier than those in the quietest places. In May–June, however, when birds started singing earlier in relation to sunrise and noise had a much weaker effect on the onset of the dawn chorus, the maximum difference in singing onset time was only 40 min. Thus, our data suggest that anthropogenic effects can reduce the behavioural synchronization of bird populations.

The observed difference in the onset of the dawn chorus between seasons was unexpected and we can only speculate as to why there was such marked seasonal effect. Singing behaviour, and specifically the dawn chorus in birds, is associated with territory defence and mate attraction and retention [12]. Thus, there is often a correlation between breeding stage and singing behaviour. Rufous-collared sparrows in the equatorial tropics use cues such as food availability and weather to time their breeding season [31,37]. There was no clear difference in meteorological conditions between the two sampling seasons [42]. In addition, we have no information about the breeding status of the recorded individuals. Anecdotal observations from the city of Bogota indicate more fledgling rufous-collared sparrows in Bogota in February and June than at other times of the year, suggesting potential breeding peaks in mid-January and mid-May (M Hernandez 2015, personal communication). A similar pattern has been observed in other parts of the Colombian Andes [38]. A breeding peak in May could indeed be accounted for by our observation that urban sparrows started to sing earlier in relation to sunrise in May–June compared to November–December,

In conclusion, our study highlights that general ecological patterns, such as latitude, can have fundamental effects on how animals respond to urbanization. Bird species like rufous-collared sparrows, which are present in both tropical and temperate zones, are suitable candidate species for further comparative studies addressing the effects of seasonal variation in day length on mating behaviours and how anthropogenic disturbances may differentially affect populations at different latitudes.

## Supplementary Material

Electronic supplementary material: Song spectrograms, song and noise spectra, variation of light and noise levels at the sampled sites, map of sampled sites, comparison rural/urban sites

## Data accessibility

Data available from the Dryad Digital Repository: http://dx.doi.org/10.5061/dryad.j23g3.
