# Supplementary material for: Anthropogenic noise, but not artificial light levels predicts song behaviour in an equatorial bird
Source: R Soc Open Sci. 2016 Jul 6;3(7):160231. doi: 10.1098/rsos.160231 (PMC4968470; doi:10.1098/rsos.160231)
Supplement: Electronic supplementary material: Song spectrograms, song and noise spectra, variation of light and noise levels at the sampled sites, map of sampled sites, comparison rural/urban sites [file rsos160231supp1.pdf]

Anthropogenic noise, but not artificial light levels predicts song behaviour in an equatorial bird

Adriana M. Dorado-Correa[1,2], Manuel Rodríguez-Rocha[2], Henrik Brumm[1]

[1] *Communication and Social Behaviour Group, Max Planck Institute for Ornithology, Seewiesen, Germany*
[2] *Foundation Chimbilako, Carrera 34 # 10 – 77, Bogotá, Colombia*

Electronic Supplementary Material

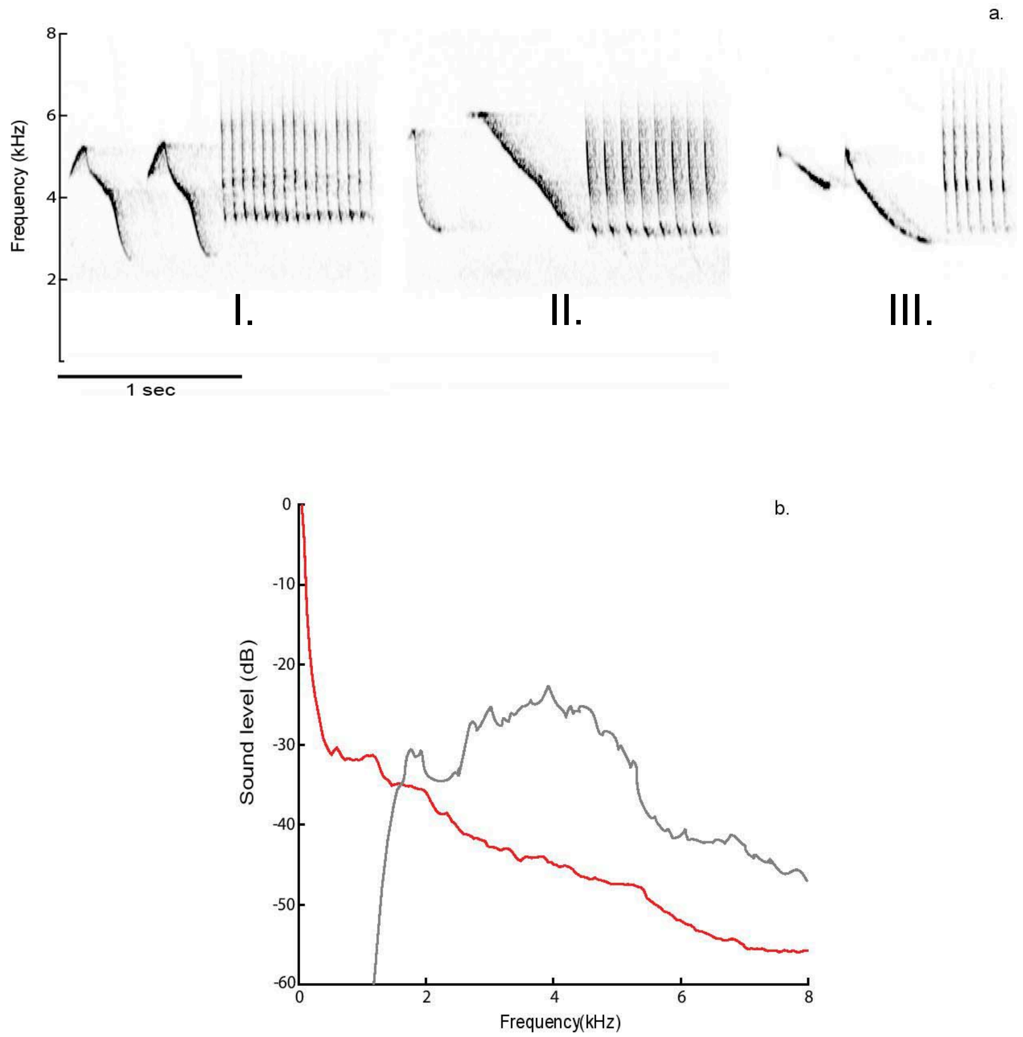

Figure S1. Rufous-collared sparrow song and exemplary traffic noise recorded in the city of Bogota. (a) spectrograms of three rufous-collared sparrows songs (I,II & III) and (b) power spectra of the average of the song of the three individuals (grey line) and the traffic noise (red line). The recordings were made with a Zoom H1 Ultra-Portable Digital Audio Recorder with a sample rate of 44.1 kHz and 16 bit accuracy.

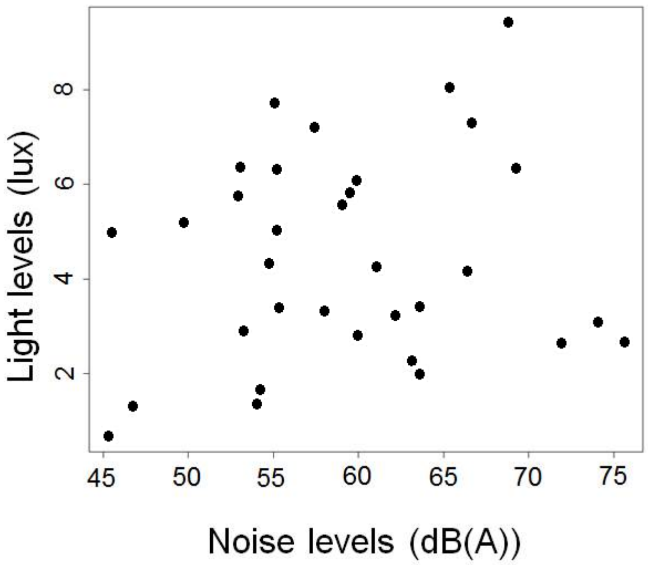

Figure S2. Noise levels and light pollution levels at the sample sites. We systematically chose locations with different combinations of light and noise and the two measures were not correlated.

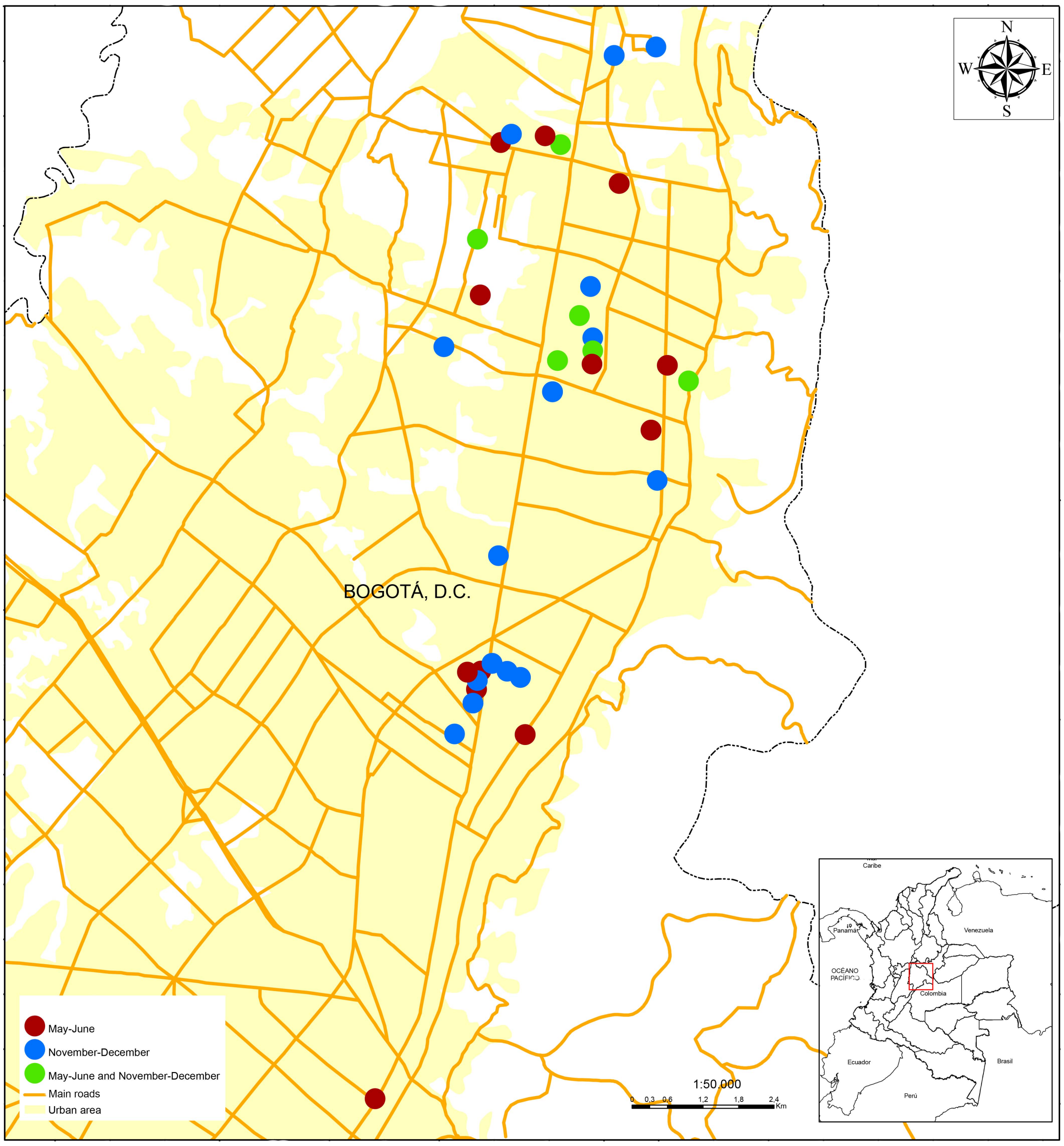

Figure S3. Bogota is the capital of Colombia, located at the centre of the country (map on the right lower corner). The black lines denote the limits of the city. The red points show to the sites visited in May-June, the blue points correspond to sites visited in November-December, and the green points show the places that were visited in May-June 2013 and November 2015. Map made by Ana Maria Bastidas-Urrutia.

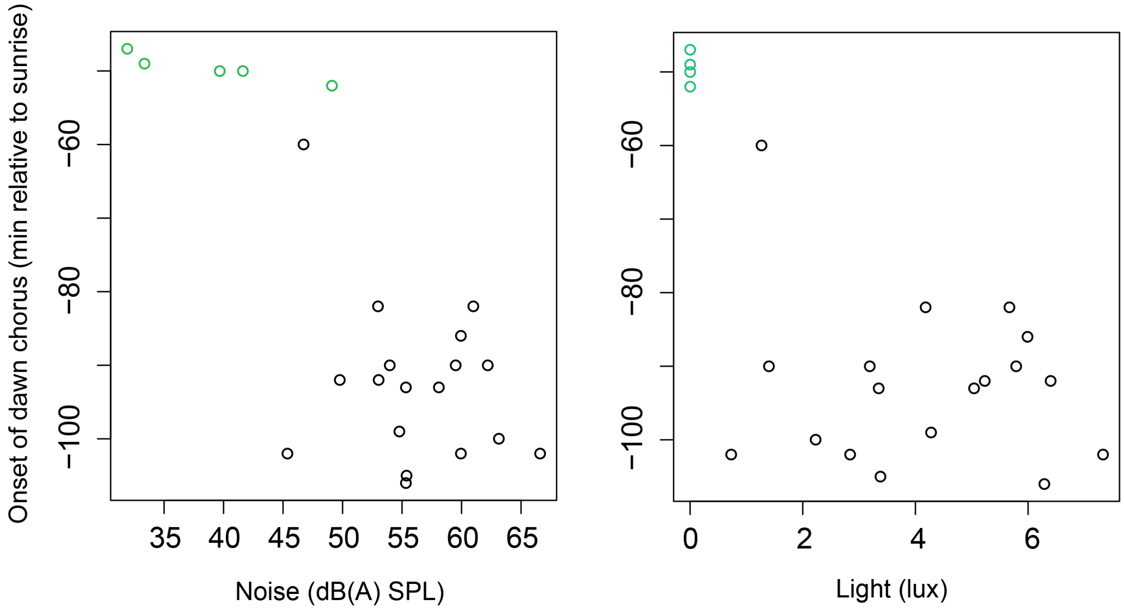

Figure S4. Onset time of the dawn chorus of rufous-collared sparrows in May-June in the city of Bogota (black) and rural areas in the Colombian Andes (green). (a) Relationship with noise levels and (b) light pollution levels.
